# A nonspecific synergistic effect of biogenic silver nanoparticles and biosurfactant towards environmental bacteria and fungi

**DOI:** 10.1007/s10646-018-1899-3

**Published:** 2018-02-06

**Authors:** Chojniak Joanna, Libera Marcin, Król Ewa, Płaza Grażyna

**Affiliations:** 1 Institute for Ecology of Industrial Areas, Environmental Microbiology Unit, 40-844 Katowice, Kossutha 6, Poland; 2 Institute of Chemistry, University of Silesia in Katowice, Szkolna 9, Katowice, 40-006 Poland; 3 Department of Phytopathology and Mycology, University of Life Sciences, 20-069 Lublin, Poland

**Keywords:** Biogenic silver nanoparticles, Biosurfactants, Antimicrobial properties, DNA

## Abstract

The present study focused on the evaluation of a nonspecific synergistic effect of biogenic silver nanoparticles (AgNPs) in combination with biosurfactants against environmental bacteria and fungi. The AgNPs were synthesized in the culture supernatants of the biosurfactant producer *Bacillus subtilis* grown in brewery effluent, molasses or Luria-Bertani media. Antibacterial activities were tested against Gram-positive and Gram-negative bacteria, while the antifungal activity was tested against phytopathogens. The interactions between biogenic AgNPs and DNA were investigated using a cryo-TEM technique. The presence of biosurfactant significantly increased the stability of biogenic AgNPs and enhanced their antimicrobial activities. The physical properties and antimicrobial activity of biogenic AgNPs were compared with chemically synthesized Ag nanoparticles. Biogenic silver nanoparticles showed a broad spectrum of activity against bacteria and fungi. They were most active against phytopathogenic fungi and Gram-positive bacteria and less active against Gram-negative bacteria. The nonspecific synergistic effect of biogenic AgNPs and biosurfactant on the phytopathogenic fungi was especially observed. In this report, the new roles of biosurfactants as a biogenic AgNPs stabilizer and enhancer of their antimicrobial properties are presented. Our results revealed that the biologically synthesized AgNPs by the biosurfactant-producing bacterium *Bacillus subtilis* grown on agro-industrial wastes, such as molasses and brewery effluent, could be used as a promising new nanoagent against microbes.

## Introduction

Nanobiotechnology (bionanotechnology, nanobiology) has recently emerged as an area of interest a result of an active integration between microbial biotechnology and nanotechnology. During the past decade, the biosynthesis of metal nanoparticles (MeNPs) has been developed as an alternative, environmentally benign nanobiotechnological method. The biological methods used to synthesize nanoparticles, often called “green-synthesis” or “green chemistry” procedures, belong to a new, green generation processes that are eco-friendly and have been designed as credible alternatives to chemical and physical methods (Thakkar et al. 2010).

Recognizing the importance of developing eco-friendly methods for the synthesis of biologically active nanoparticles, scientists have recently started looking into research relating to the synthesis of metallic nanoparticles with the additional use of biosurfactants as capping agents (Kiran et al. 2010; Reddy et al. 2009a; Reddy et al. 2009b; Singh et al. 2011). It was observed that biosurfactants produced by microorganisms can play a very important role in the process of metallic nanoparticle aggregation and stabilization. One of the biosurfactant modes of action is through adsorbing onto metallic nanoparticles (MeNPs), surface-stabilizing the nanoparticles and preventing subsequent aggregation (Chen and Yeh 2002; Kvitek et al. 2008).

Various strains of *Bacillus* produce a broad spectrum of biosurfactants, primarily lipopeptides from the surfactin, iturin and fengicin families. There are several reports on using *Bacillus* bacteria and their biosurfactant production for controlling bacterial, mould, and fungal pathogens (Joshi et al. 2008; Rai et al. 2009; Singh et al. 2011). Recent studies have confirmed that specially formulated metal nanoparticles have a good antimicrobial activity and that nanoparticle-based antimicrobial formulations could be effective bactericidal and fungicidal materials (Franci et al. 2015; Guzman et al. 2012; Krishnaraj et al. 2012; Martinez-Gutirrez et al. 2010; Rai et al. 2009). Investigations of the biological activity of AgNPs is important to further their potential applications, and the antimicrobial activities of nanoparticles in combination with biosurfactants are still unclear.

In the current study, a nonspecific synergistic effect of biogenic silver nanoparticles (AgNPs) and biosurfactant produced by *Bacillus subtilis* towards environmental bacteria and fungi was investigated. The effect of biologically and chemically synthesized AgNPs on the bacteria and phytopathogenic fungi was also compared.

## Materials and methods

### Characterization and culture of *B. subtilis* strain I’-1a

The biosurfactant-producing strain used in this study was identified as *Bacillus subtilis* I’-1a based on its biochemical properties and 16S rRNA gene sequence analysis, and its biosurfactants production was also described (Bernat et al. 2016; Płaza et al. 2015).

The bacterial cultures were prepared as described by Płaza et al. (2015) and then grown aerobically on brewery waste, molasses or Luria-Bertani media at 30 °C for 96 h with constant shaking (110 rpm) (Innova 42 Incubator, New Brunswick Scientific, USA). After culturing, the freshly grown bacterial culture was centrifuged at 10,000*g* (Eppendorf) for 10 min and the supernatant was collected and filtered through sterile 0.22 µm filter into sterile flasks. Cell-free supernatant was used to synthesize AgNPs.

### Biological and chemical synthesis of AgNPs

Biological synthesis of AgNPs was done according to Płaza et al. (2016). The bioreduction of silver ions was monitored by UV-Vis spectrophotometer (Lange DR5000 with a resolution of 0.72 nm) as a function of time. During the synthesis, a colour change was observed from yellow to dark brown. The chemical synthesis of silver nanoparticles without the biosurfactant was performed as a control for the biological synthesis. The chemical synthesis of AgNPs was carried out as described by Mendrek et al. (2016).

In the experiments, the concentration of biological and chemical AgNPs was evaluated by atomic absorption spectroscopy (AAS). Biologically and chemically synthesized AgNPs were applied at a concentration of approximately 165 mg/L.

### Evaluation of the effect of AgNPs on bacteria and fungi

The bacterial strains used in this experiment were isolated from the test fields at PIA (Development and Assessment Institute in Waste Water Technology, RWTH Aachen Germany) from a fluidized bed bioreactor as an example of onsite wastewater treatment plants. The isolation and identification of the bacterial strains were described by Jałowiecki et al. (2017). Most of the selected bacteria were multi-antibiotic resistant.

The fungal plant pathogens studied originated from the collection of the Department of Phytopathology and Mycology, University of Life Sciences in Lublin. The following phytopathogens that were isolated from various parts of caraway, angelica and grapevines were used: *Alternaria alternate*, *Boeremia strassesi*, *Colletotrichum dematium*, *Colletotrichum fuscum*, *Cylindrocarpon destructans, Diaporthe eres, Diplocereus hypericinum, Fusarium equiseti, Fusarium oxysporum, Phylloticta plantagnus, Rhizoctonia solani, Sclerotinia sclerotiorum, Fusarium avenaceum, Fusarium avenaceum*. The majority of these pathogens can infect many plant species but some of them, such as *Alternaria alternate, Colletotrichum fuscum* and *Fusarium oxysporum*, only infect one host or a few species within the same botanical family. These fungi were grown on Potato Dextrose Agar (PDA) at room temperature for further experimentation.

The antibacterial activities of the Ag nanoparticles to environmental strains were determined by the agar disk diffusion method. The plates were swabbed with 100 μL of bacterial culture with an optical density of 0.5 on the McFarland scale, then the sterile disks were placed on the surface. 10 µL of the biological and chemical AgNPs solutions of the concentration of 165 mg/L was put on the discs. The final concentration of silver nanoparticles was around 0.00165 mg/L per disc. The plates were incubated at 30 °C for 24–48 h, and the size of the inhibition zones was recorded. The chemically synthesized nanoparticles without biosurfactant was used as a control in all the experiments performed.

The in vitro assay for antifungal activity was performed on PDA fungal growth treated with synthetized silver nanoparticles. 100 µL silver nanoparticles solution was distributed on the media plates, then a 5 mm diameter disc of fungal mycelium was put on the center of the plate.

Each assay was carried out in triplicate and the results are presented as the mean values.

### Interaction of prepared AgNPs with DNA by cryogenic transmission electron microscopy (Cryo-TEM)

Chromosomal DNA from the reference strains *Escherichia coli* ATCC 25922 and *Bacillus subtilis* ATCC 6633 was isolated using the standard protocol of the Roche High Pure PCR Template Preparation kit (Ref. 11 796 828 001). To study the interaction between the DNA and the synthesized AgNPs, 5 µL of bacterial DNA at a concentration of 218 µg/mL was mixed with 10 µL of a solution of silver nanoparticles that had been synthesized by chemical or biological methods. Cryogenic transmission electron microscopy images (cryo-TEM) were obtained using a Tecnai F20 TWIN microscope (FEI Company, USA) equipped with a field emission gun, which operates at an acceleration voltage of 200 kV. Images were recorded on an Eagle 4k HS camera (FEI Company, USA) and processed with TIA software (FEI Company, USA). A 2 µL sample drop was placed on a holey carbon-coated film supported by a copper grid (Quantifoil R 2/2; Quantifoil Micro Tools GmbH, Germany), gently blotted with filter paper and then was immediately frozen in liquid ethane using a fully automated Vitrobot Mark IV (FEI Company, USA) blotting device. Prior to use, the grids were activated for 15 s in a PELCO glow discharge system (Ted Pella Inc. USA). After preparation, the vitrified specimens were kept under liquid nitrogen until they were inserted into a Gatan 626 cryo-TEM-holder (Gatan Inc., USA) and analysed in the TEM at −178°C. The pictures were processed using ImageJ software.

## Results and discussion

**Fig. 1:**
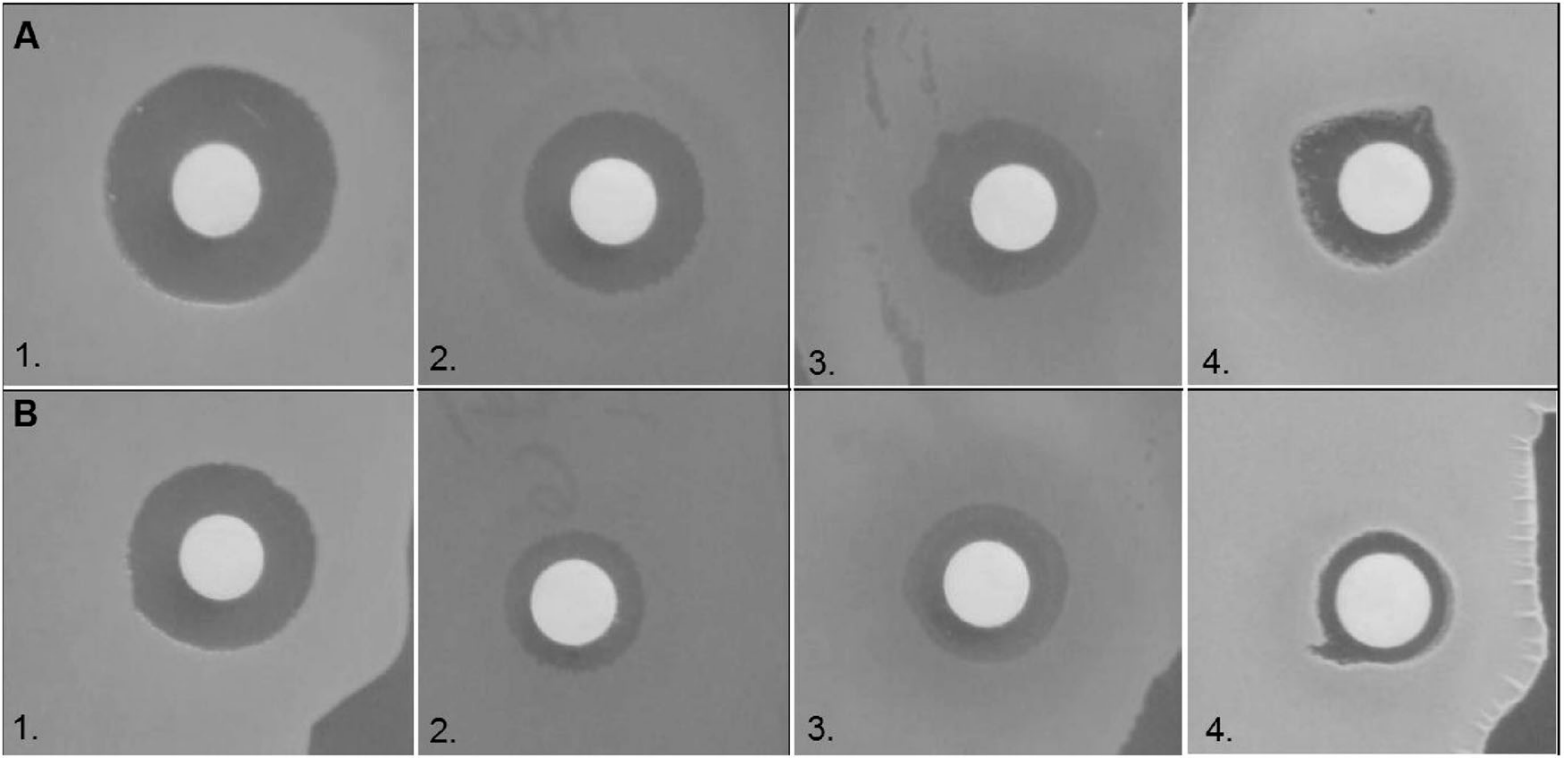
Antibacterial activity of biogenic AgNPs (**a**) and chemically synthesized AgNPs (**b**). 1. Micrococcus luteus; 2. Bacillus subtilis ACTT 6633; 3. Bordetella petri; 4. Mycobacterium flavenscens

The physical properties of the AgNPs formed by bio- and chemical synthesis have been discussed by Mendrek et al. (2016). The results of antibacterial activities of biogenic AgNPs against various bacteria are reported in Table 1. AgNPs showed reliable activity against the tested bacterial strains, with different degrees of efficacy. The antibacterial activity of biosynthesized AgNPs was found to be highly effective against Gram-positive bacteria, whereas the antibacterial activity against Gram-negative was found to be mild. The activity against *Pseudomonas fluorescens* and *Ralstonia picketti* was not exhibited. The highest antimicrobial activity of the biogenic AgNPs was observed against *Paenibacillus bercinonensis* and *Micrococcus luteus* (Fig. 1a) The maximum zones of inhibition diameters were 14 and 16 mm for *Paenibacillus bercinonensis* and *Micrococcus luteus*, respectively. AgNPs produced in all media supernatants showed the highest inhibition against the reference strain *Bacillus subtilis* ATCC 6633 compared to the other Gram-positive bacteria tested. The *Bacillus subtilis* ATCC 6633 reference strain was the most sensitive to silver nanoparticles that were synthesized biologically. The difference in antimicrobial activity between Gram-positive and Gram-negative bacteria could be due to their differences in membrane structure. Gram-positive bacteria have a thick peptidoglycan layer, whereas the peptidoglycan layer in the Gram-negative bacteria is thin but is surrounded by a lipid layer.

**Table 1:** Antibacterial activity spectrum of silver nanoparticles synthesized biologically using cultural filtrate of *Bacillus subtilis* growing on LB medium, brewery effluent or molasses. Antibacterial activities of AgNPs chemically synthesized without biosurfactant as a control are presented

Strains	Diameter of inhibition zones (mm)				
	AgNPs chemically synthesized	AgNPs synthesized on LB medium	AgNPs synthesized on brewery effluent	AgNPs synthesized on molasses	Ag^+^ (1 mM)
Gram (+)					
*Micrococcus luteus* B	12.2 ± 0.079	12.18 ± 0.181	11.22 ± 0.192	10.51 ± 0.182	7.5 ± 0.017
*Paenibacillus bercinonensis*	10.27 ± 0.019	14.96 ± 0.049	14.37 ± 0.109	12.01 ± 0.634	0.00
*Micrococcus luteus*	11.53 ± 0.262	9.05 ± 0.207	10.63 ± 0.069	15.95 ± 0.033	14.00 ± 0.01
*Microbacterium testaceum*_1	7.51 ± 0.028	11.45 ± 0.073	12.11 ± 0.124	9.04 ± 0.188	0.00
*Microbacterium testaceum*_2	17.18 ± 0.207	10.54 ± 0.423	11.41 ± 0.503	10.02 ± 0.059	0.00
*Bacillus subtilis* ATCC 6633	10.86 ± 0.164	11.07 ± 0.065	16.07 ± 0.205	18.91 ± 0.521	10.5 ± 0.28
Gram (−)					
*Pseudomonas fluorescens* biotype G	15.97 ±± 0.124	10.61 ± 0.068	9.16 ± 0.069	17.59 ± 0.154	0.00
*Bordetella bronchiseptica*	8.95 ± 0.102	9.03 ± 0.169	10.43 ± 0.083	10.71 ± 0.219	10.25 ± 0.14
*Bordetella petrii*	12.40 ± 0.32	13.25 ± 0.032	12.60 ± 0.028	14.80 ± 0.064	12.5 ± 0.17
*Pseudomonas fluorescens* _1	0.00	0.00	0.00	0.00	0.00
*Pseudomonas fluorescens* 2	13.36 ± 0.126	0.00	0.00	0.00	10.00 ± 0.23
*Ralstonia picketti*	10.07 ± 0.005	0.00	0.00	0.00	10.75 ± 0.02
*Mycobacterium flavenscens*	7.06 ± 0.013	7.35 ± 0.074	9.18 ± 0.007	10.18 ± 0.44	0.00
*Serratia marcescens*	6.93 ± 0.085	7.90 ± 0.134	8.03 ± 0.154	7.66 ± 0.376	6.24 ± 0.26
*Escherichia coli* JM 109	9.09 ± 0.587	8.09 ± 0.73	8.96 ± 0.039	8.36 ± 0.019	12.00 ± 0.02
*Escherichia coli* ATCC 25922	6.40 ± 0.014	8.86 ± 0.05	7.11 ± 0.071	7.20 ± 0.02	11.00 ± 0.08

Determinations were performed in triplicate and data correspond to mean values ± standard deviations

Chemically synthesized AgNPs were also more active against Gram-positive bacteria (Fig. 1b). The diameters of the measured inhibition zones were larger for Gram-positive bacteria. The average size of the inhibition zones for *Microbacterium testaceum* was 17.2 mm. To summarize, the microbial activity of chemically synthesized AgNPs was lower compared to biogenic AgNPs.

Guzman et al. (2012) obtained similar results to those presented in this study. They evaluated the antibacterial activity of chemically synthesized silver nanoparticles against Gram-positive and Gram-negative bacteria. The nanoparticles showed reasonable activity against the tested bacteria, however, the inhibition zones for the Gram-positive bacteria were larger compared to Gram-negative bacteria.

The antimicrobial action of AgNPs is linked with four mechanisms as proposed by Dakal et al. (2016): (1) AgNPs attach to the surface of the cell wall and the membrane and disturb its cellular functions, such as respiration and permeability, (2) AgNPs penetrate inside the cell, and damaging intracellular structures and interacting with sulfur- and phosphorus-containing biomolecules such as DNA or proteins, (3) AgNPs induce cellular toxicity and oxidative stress cause by the generation of reactive oxygen species and free radicals, and (4) AgNPs modulate signal transduction pathways. Generally, the antibacterial activity of biologically synthesized AgNPs was enhanced through their stabilization and the presence of biosurfactants in cell-free supernatants used in the experiment (Kiran et al. 2010). The silver nanoparticles were stabilized by biosurfactants produced by *Bacillus subtilis*. Previously, we described the bacterial strain *B. subtilis* I’-1a as a producer of surfactin, iturin and fengycin (Bernat et al. 2016; Płaza et al. 2015). It was also found that lipopeptides extracted from the supernatant have a strong antimicrobial effect on uropathogenic bacteria, including effects on planktonic growth, and the processes of biofilm formation and dislodging (Moryl et al. 2015). The extract obtained from strain I’-1a exhibited a greater inhibitory effect against both planktonic and sessile forms of *Escherichia coli*, *Serratia marcescens*, *Enterobacter cloacae*, *Proteus mirabilis*, *Citrobacter freundii* and *Enterococcus faecalis*.

**Fig. 2:**
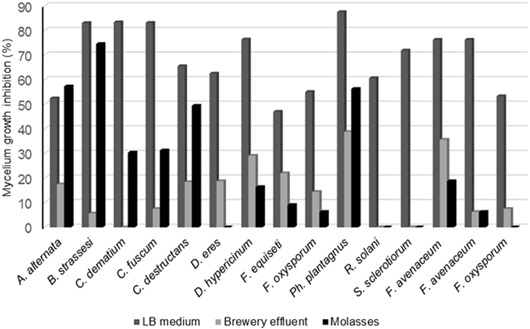
Fungal mycelium growth inhibition expressed as antifungal index (%)

**Fig. 3:**
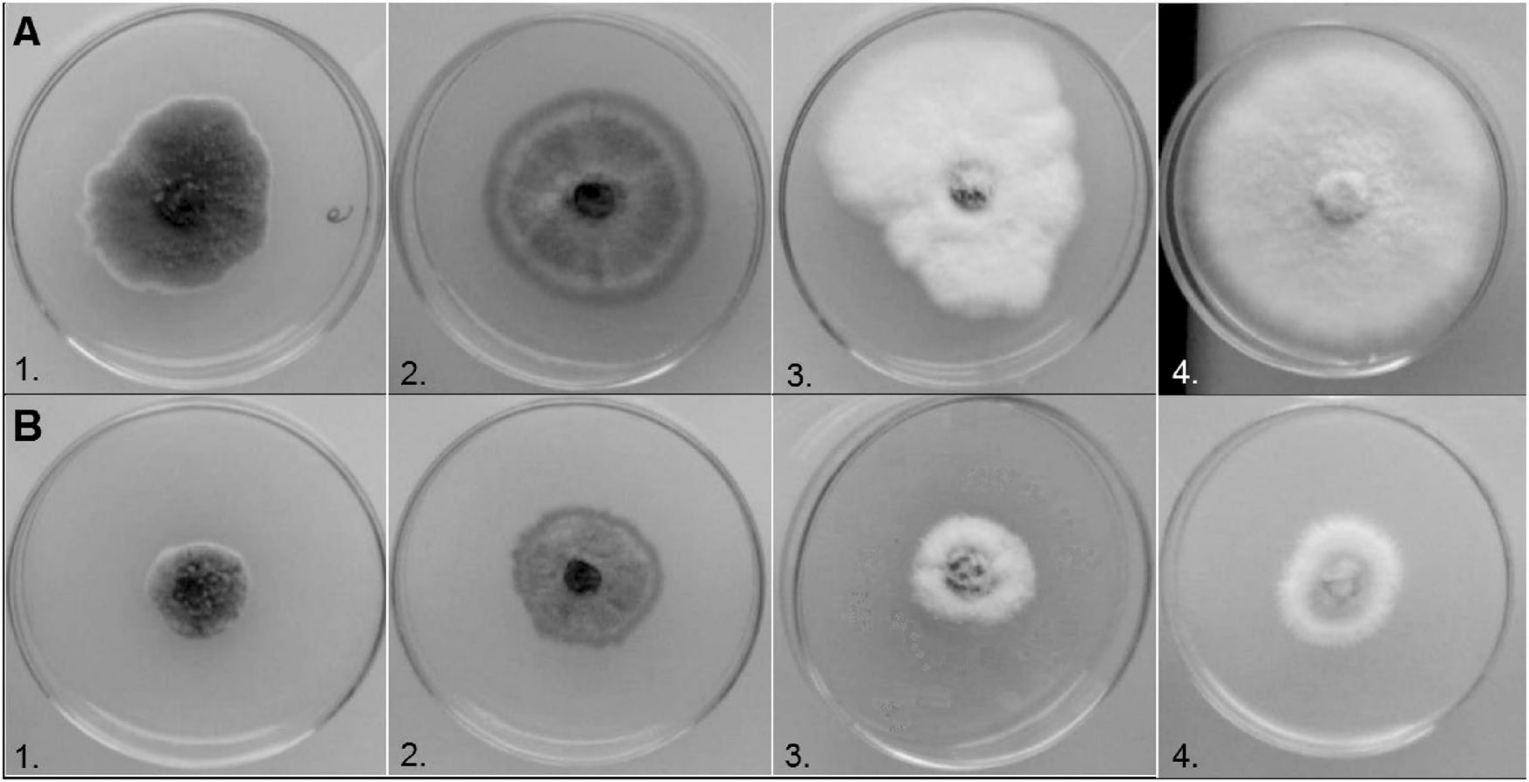
The effect of biogenic AgNPs on the fungal growth. (**a**) Growth of fungi on PDA medium (control). (**b**) Growth of fungi on PDA medium with biogenic AgNPs. The AgNPs were synthetized in the cell-free supernatant obtained from the culture of *Bacillus* strain growing on LB medium. 1. Alternaria alternata; 2. Colletotrichum dematium; 3. Phylloticta plantagnus and 4. Fusarium oxysporum

We observed significant antifungal activity of AgNPs that were biologically synthesized against various fungal strains (Figs. 2 and 3). A greater activity was observed for AgNPs synthesized in cell-free supernatants of a bacterial LB culture when molasses and brewery effluent culture supernatants were used. Mycelial growth inhibition above 50% was observed for 13 fungal species (Fig. 2). In the Fig. 3 the effect of biogenic AgNPs on fungal growth is presented. The nonspecific combined effect of biogenic AgNPs and biosurfactant was observed. In contrast, very low antifungal activity was observed for chemically synthesized AgNPs and Ag^+^ ions. In this case, no effect antifungal activity against 10 selected fungi was observed.

Donio et al. (2013) reported that *Bacillus sp*. BS3 biosurfactants possess an important antifungal activity against different pathogens. Similar results were obtained in our previous research conducted by Płaza et al. (2012). The antifungal activity of various extracts from cultures of *Bacillus* strains was evaluated, including *Bacillus subtilis* I’-1a growing on agro-industrial wastes. In the present study, the antifungal activity of biogenic AgNPs in combination with biosurfactant obtained was much higher. The mycelium growth rate inhibition was above 50% for 13 of the tested fungal strains, while no effect of chemically synthesized AgNPs was observed on the most of the tested fungi. AgNPs modified with different coatings, such as biosurfactants, generally show an increased antibacterial and antifungal activities that could be related to the increased uptake as a consequence of a greater binding ability of nanoparticles to the cells (Kvitek et al. 2008).

**Fig. 4:**
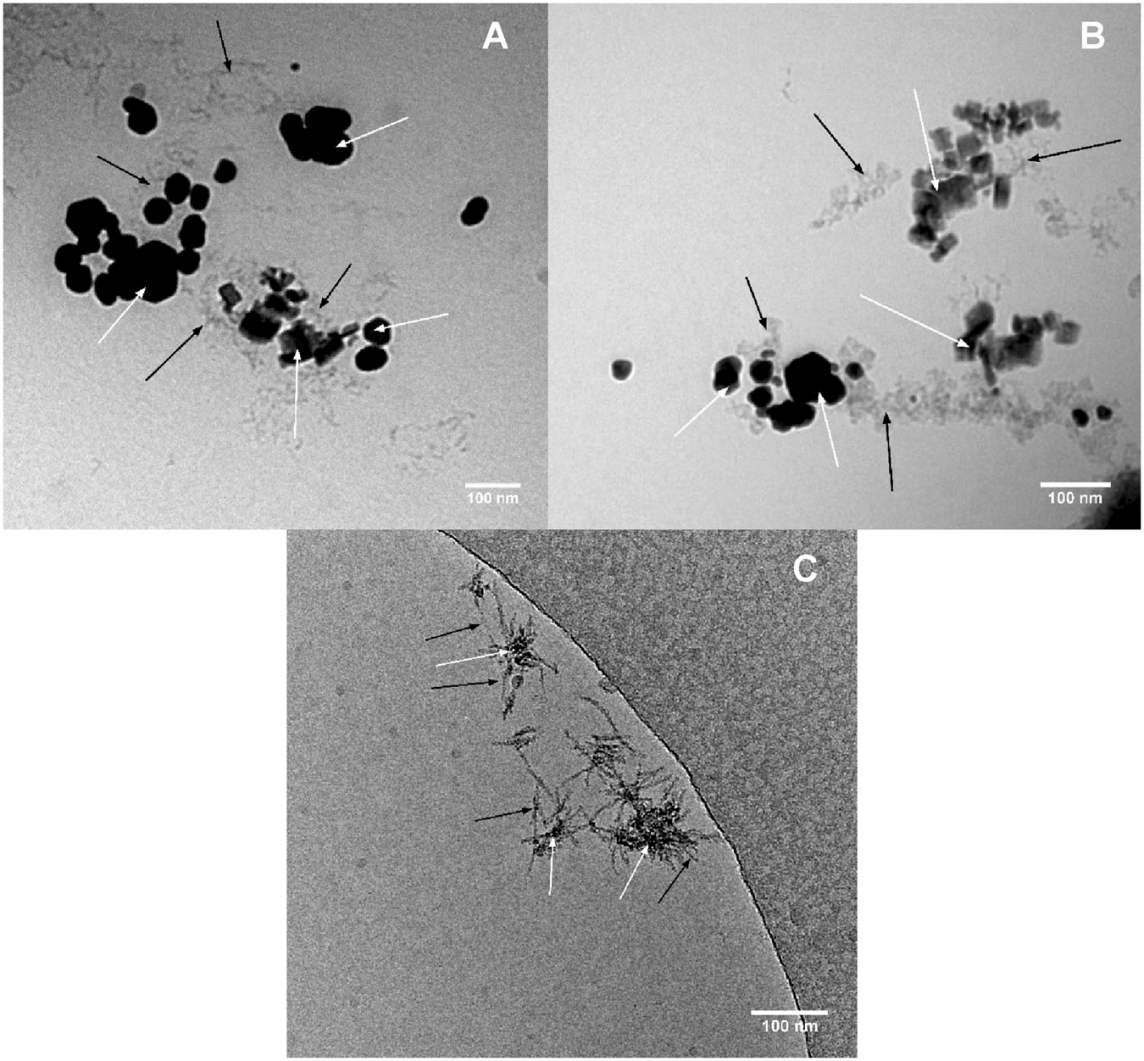
Cryogenic transmission electron micrographs obtained for DNA-AgNPs structure formed by chemical nanosilver (**a, b**) and biological nanosilver (**c**). The white arrow indicates silver nanoparticles, the black arrow indicates DNA fragments of E. coli ATCC 25922 DNA

The structures formed by bacterial DNA with Ag nanoparticles prepared through chemical or biological protocols were also evaluated. Information about the morphologies of structures formed by DNA and silver nanoparticles are provided in Fig. 4. Micrographs indicated that the silver nanoparticles prepared through biological methodology are significantly lower (Fig. 4c) than those prepared by chemical protocol (Fig. 4a,b). DNA particles were observed to be densely accumulated around the biogenic AgNPs (Fig. 4c). One of the potential mechanism might be strong affinity between metal nanoparticles and nitrogen bases with the hydrogen bonds between matched sequences (Mishra et al. 2012).

All the factors which influence the activity of AgNPs (concentration, size, shape, and their use in combination with various agents such as antibiotics and biosurfactants) should be taken into consideration when preparing AgNPs for application purposes. Rai et al. (2012) described the effect of combinations of silver nanoparticles and antibiotics on bacteria, as well as the fungicidal activity of nanoparticles alone and in combination with the commercially available antifungal agent fluconazole.

The antimicrobial potential of AgNPs can be increased by manipulating the size, physio-chemical properties and use in combination with various chemicals such as biosurfactants.

## Conclusions

Biologically produced AgNPs have very specific characteristics that are unobtainable by conventional chemistry.

In conclusion, the antimicrobial activity of silver nanoparticles shows that they have great potential to be used as antimicrobial agents against various bacteria and fungi.

The current study suggested that the synergistic effect of biosurfactant in conjugation with biologically synthesized AgNPs increased the susceptibility of tested bacteria and fungi. Thus, the bacterial system of AgNP production has the potential for the low-cost and environmentally friendly production of silver nanoparticles with higher antimicrobial potential.

Biogenic silver nanoparticles showed a broad spectrum of antimicrobial activity and were most active against fungi and Gram-positive bacteria, and less active against Gram-negative bacteria. The synergistic effect of AgNPs and biosurfactant, especially against phytopathogens, was observed. The combination of biogenic AgNPs with the biosurfactant increased the antibacterial and antifungal activities. In this paper, the new roles of biosurfactants as stabilizers of biogenic AgNPs and enhancers of their antimicrobial properties are presented.
